# Kinetics of anti-nucleocapsid IgG response in COVID-19 immunocompetent convalescent patients

**DOI:** 10.1038/s41598-022-16402-0

**Published:** 2022-07-20

**Authors:** Mariam Movsisyan, Armine Chopikyan, Irina Kasparova, Gohar Hakobjanyan, Fabrice Carrat, Meline Sukiasyan, Marieta Rushanyan, Mariam Chalabyan, Sanobar Shariff, Burhan Kantawala, Anna Keshishyan, Alvard Hovhannisyan, Armine Hakobyan, Gayane Petrosyan, Armen Minasyan, Armen Muradyan, Arsene Mekinian, Konstantin Yenkoyan

**Affiliations:** 1 Department of Allergology and Clinical Immunology, Yerevan State Medical University named after Mkhitar Heratsi, Yerevan, Armenia; 2 Department of Public Health and Healthcare Organization, Yerevan State Medical University named after Mkhitar Heratsi, Yerevan, Armenia; 3 Department of Histology, Yerevan State Medical University named after Mkhitar Heratsi, Yerevan, Armenia; 4 Laboratory-Diagnostic Center of Heratsi Clinical Hospital, Yerevan State Medical University named after Mkhitar Heratsi, Yerevan, Armenia; 5 Sorbonne Université, Inserm, Institut Pierre-Louis d’Epidémiologie et de Santé Publique, Paris, France; 6 Département de Santé Publique, APHP, Sorbonne Université, Paris, France; 7 Yerevan State Medical University named after Mkhitar Heratsi, Yerevan, Armenia; 8 Department of Infectious Diseases, Yerevan State Medical University named after Mkhitar Heratsi, Yerevan, Armenia; 9 Service de Médecine Interne et Inflammation-Immunopathology-Biotherapy Department (DMU i3), Sorbonne Université, AP-HP, Hôpital Saint Antoine, 75012 Paris, France; 10 French-Armenian Clinical Research Center, 0051 Yerevan, Armenia; 11 Department of Biochemistry, Yerevan State Medical University named after Mkhitar Heratsi, Yerevan, Armenia; 12 Neuroscience Laboratory, Cobrain Center, Yerevan State Medical University named after Mkhitar Heratsi, Yerevan State Medical University named after Mkhitar Heratsi, Yerevan, Armenia

**Keywords:** Immunology, Infectious diseases

## Abstract

The comprehension of a long-term humoral immune response against SARS-CoV-2 can shed light on the treatment and vaccination strategies of COVID-19 disease, improving the knowledge about this virus infection and/or re-infection. We assessed the IgG antibodies against SARS-CoV-2 nucleocapsid (N) protein (anti-SARS-CoV-2 (N) IgG) in 1441 COVID-19 convalescent patients within 15 months longitudinal study from middle-developed country. The main inclusion criteria was positive RT– PCR result on nasopharyngeal swab samples at least one month before antibody testing and absence of any induced or inherited immunodeficiency. 92.7% of convalescent patients’ serum contained anti-SARS-CoV-2 (N) IgG and only 1.3% of patients had a delayed antibody response. In the majority of convalescent patients’ the durability of antibodies lasted more than one year. The kinetics of anti-SARS-CoV-2 (N) IgG took a bell-shaped character—increased first 25–30 weeks, then started to decrease, but were still detectable for more than 15 months. We found that on the one hand anti-SARS-CoV-2 humoral response level correlates with disease severity, on the other, in particular, the level of peak antibodies correlates with age—older patients develop more robust humoral response regardless of sex, disease severity and BMI.

## Introduction

At the end of 2019, patients with viral infection symptoms and pneumonia were found in Wuhan, China, leading to the discovery of a novel severe acute respiratory syndrome coronavirus 2 (SARS-CoV-2). More than 169 million cases and 3.5 million deaths were reported from SARS-CoV-2disease up to May 28, 2021.

Apprehending the immune responses and the features of antibody production after SARS-CoV-2 are key points in developing an effective treatment. Hence, the significance of improved understanding of immune response to SARS-CoV-2 virus is indisputable. There are confirmed facts that all arms of the immune responses to SARS-CoV-2, although many questions remain uncertain^1^.

One of the central issues of the disease is the probability of reinfection and long-term immunity. A limited number of documented and confirmed cases of re-infections are registered in immunocompetent individuals^2–4^. The resistance to re-infection may be less a function of the durability of the immune response, than the peculiarities of the individual response or the breadth of immunity. During reinfection, high avidity IgG and elevated titers of neutralizing antibody were discovered. This indicates that the first infection’s priming of immunity made for a more robust antibody response in the second infection^4^.

**Figure 1:**
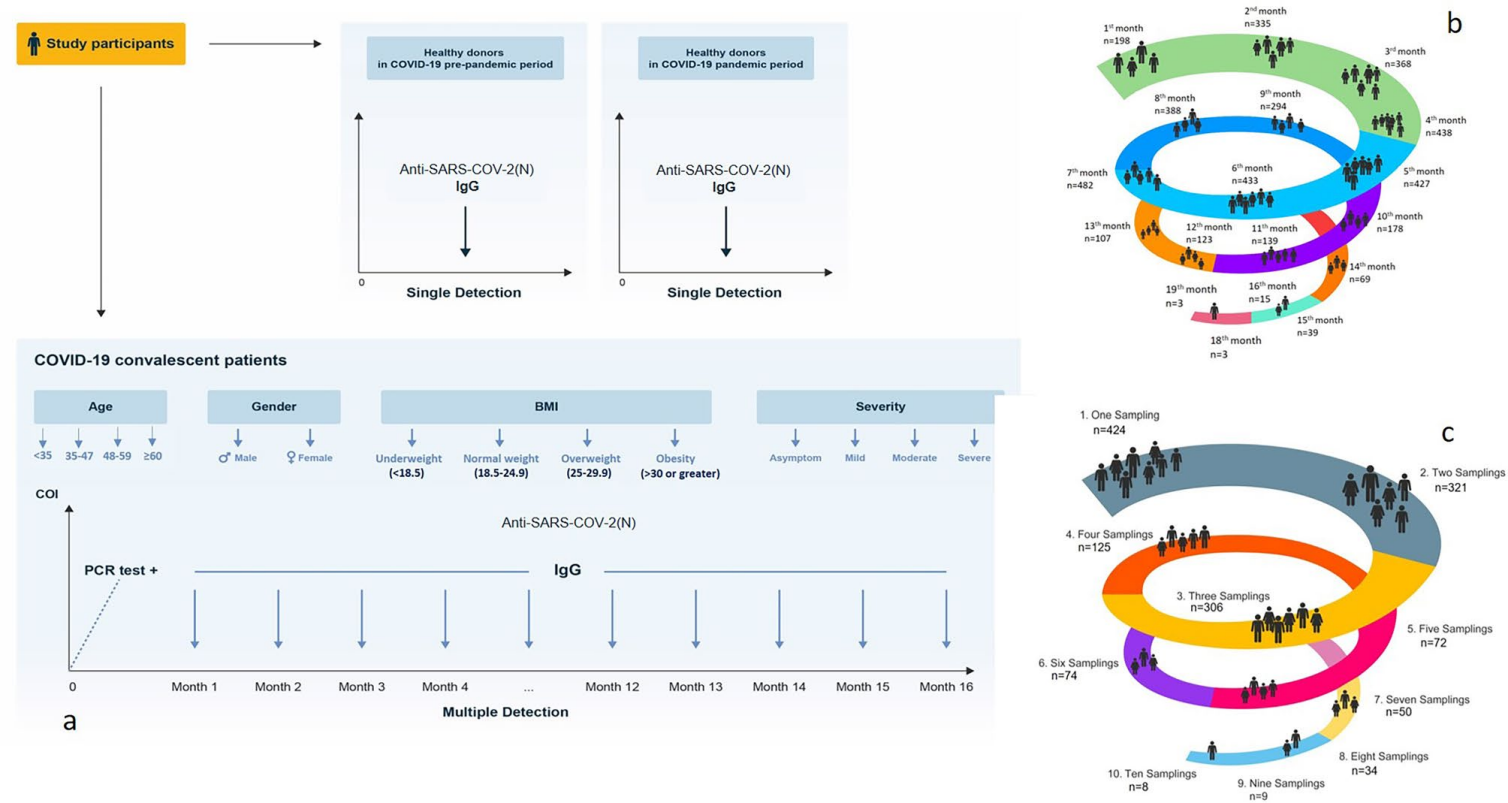
Design of the prospective study. (**a**) SARS-CoV-2 convalescent patients were involved in the study after maximum of a month of positive PCR testing, and then sampled monthly up to 19 months (*subsequent detailed analysis of antibodies titer was performed for 15 months follow-up*). Two groups of healthy donors were also included in the study: “pre-pandemic control group”—healthy donors before SARS-CoV-2 pandemic period (from 2017 up to February 2020), and “SARS-CoV-2 pandemic group” -healthy donors throughout the SARS-CoV-2 pandemic period (started from March 2 of 2020). (**b**) Number of patients’ inclusion per months. (**c**) The distribution of patients with different number of samplings for anti-SARS-Cov2 antibodies.

As it is well known, during the common human coronaviruses neutralizing antibodies are induced and last for years, providing protection from reinfection or attenuated disease, even if individuals get re-infected^5^. Long-term follow-up studies of SARS-COV showed the decline of antibody titers over 2 to 3 years, although in some patients neutralizing antibodies were detected 12 years after infection^6^. Thus, the decay in antibody production after SARS-CoV-2 infection cannot be extrapolated from early time points, demonstrating the need for longer-term follow-up studies^7^. Seroconversion and virus neutralization between 5 and 14 days after symptom onset have been well-documented, but scarce data are available about the durability of antibody production and immunity after a long period of SARS-CoV-2 infections. Contraindicating conclusions exist regarding the duration of immunity, with a rapid decay of protective antibodies within a 3–4 months^8–11^ and in opposite persistence of antibodies more than 5–6 months. Several factors have been evaluated and correlated with a higher or lower antibody response against SARS-CoV-2, such as disease severity^12–14^, BMI^12,15,16^, sex^12–14,17^ and age^12–15,18,19^, but factors associated with long term serological response are not fully evaluated. Therefore, in this study, we aimed to describe the seroprevalence and kinetics of IgG antibodies against SARS-CoV-2 nucleocapsid (N) protein (anti-SARS-CoV-2 (N) IgG) in convalescent immunocompetent patients and analyzed the factors associated with the seropositivity and the humoral response persistence (Fig. 1).

## Results

### Patients’ general characteristics

The study group included 1441 SARS-CoV-2 convalescent patients, out of which 74.1% were female (n = 1004 patients) with mean age 47 ± 15 years and BMI 27 ± 5.2 kg/m^2^. SARS-CoV-2 infection was asymptomatic in 104 cases (7.2%), mild in 846 cases (58.7%), moderate in 429 cases (29.8%) and severe in 62 cases (4.3%). The most common symptoms of SARS-CoV-2 infection were fever (n = 1089; 80.4%), fatigue (n = 924; 68.2%), headache (n = 626; 46.2%), myalgia (n = 531; 42.1%), cough (n = 467; 34.5%), loss of taste (428; 31.6%) and olfactory impairment (n = 537; 39.6%) (Table 1). The most common comorbidities were arterial hypertension (18.9%), autoimmune thyroiditis (6.3%) and diabetes mellitus (5.6%) without any cases of neoplasia or hematological disease or any immunosuppressive therapies**.** During the median follow-up of 44 weeks, only 1 (1.9%) convalescent patient developed a reinfection 12 months after of the first positive PCR testing and none received anti-SARS-CoV-2 vaccine (Supplementary Fig. 1).

**Table 1:** Demographic data of COVID-19 convalescent patients.

	Number of patients	Prevalence (%)
**Gender**		
Male	351	25.9
Female	1004	74.1
**Severity**		
Asymptomatic	104	7.2
Mild	846	58.7
Moderate	429	29.8
Severe	62	4.3
**Symptoms**		
Fever	1089	80.4
Weakness	924	68.2
Headache	626	46.2
Body pain	570	42.1
Olfactory or taste disturbance	537	39.6
Muscle pain	531	39.2
Cough	467	34.5
Dizziness	459	33.9
Loss of taste or smell	428	31.6

### Seroprevalence, seroconversion and kinetics

**Figure 2:**
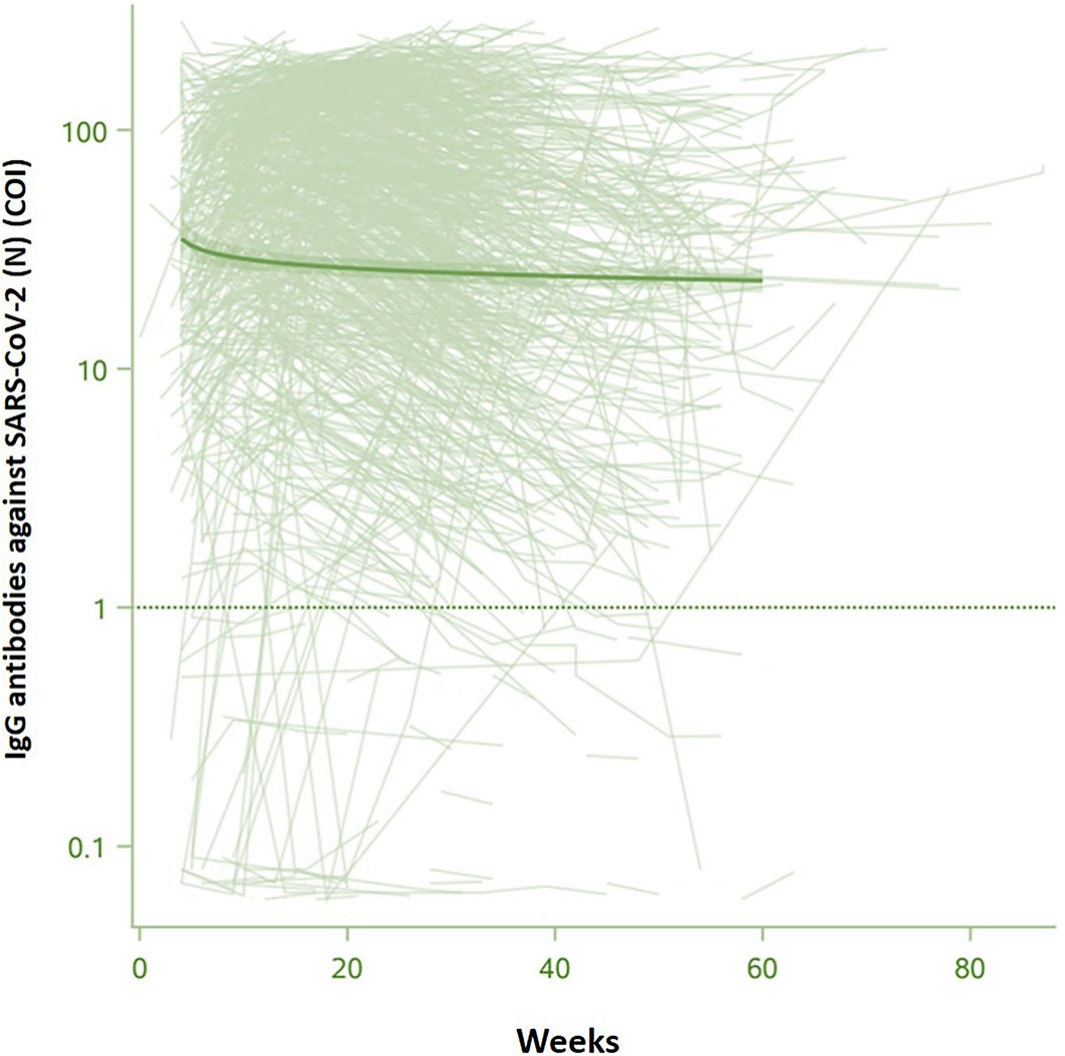
Serum IgG antibodies titers against SARS-CoV-2 nucleocapsid protein (N) of convalescent patients (n = 1441) at the different times of sampling (expressed in logarithmic expression).

Anti-SARS-CoV-2 (N) IgG measured over the time are represented in Fig. 2. The serum of 92.7% (n = 1336) of SARS-CoV-2 convalescent patients contained anti-SARS-CoV-2 (N) IgG (mean level 66.42 ± 1.04). Notably, there was a substantial interindividual variation in antibodies levels varying importantly between patients. The anti-SARS-CoV-2 (N) IgG level in convalescent patients were significantly increased in comparison to pre-pandemic and pandemic healthy controls (Supplementary Fig. 2). The median time to anti-SARS-CoV-2 (N) IgG positivity was 16 weeks (ranges 3–61) and only 13 (1.3%) patients have a delayed antibody response (Supplementary Fig. 3).

**Figure 3:**
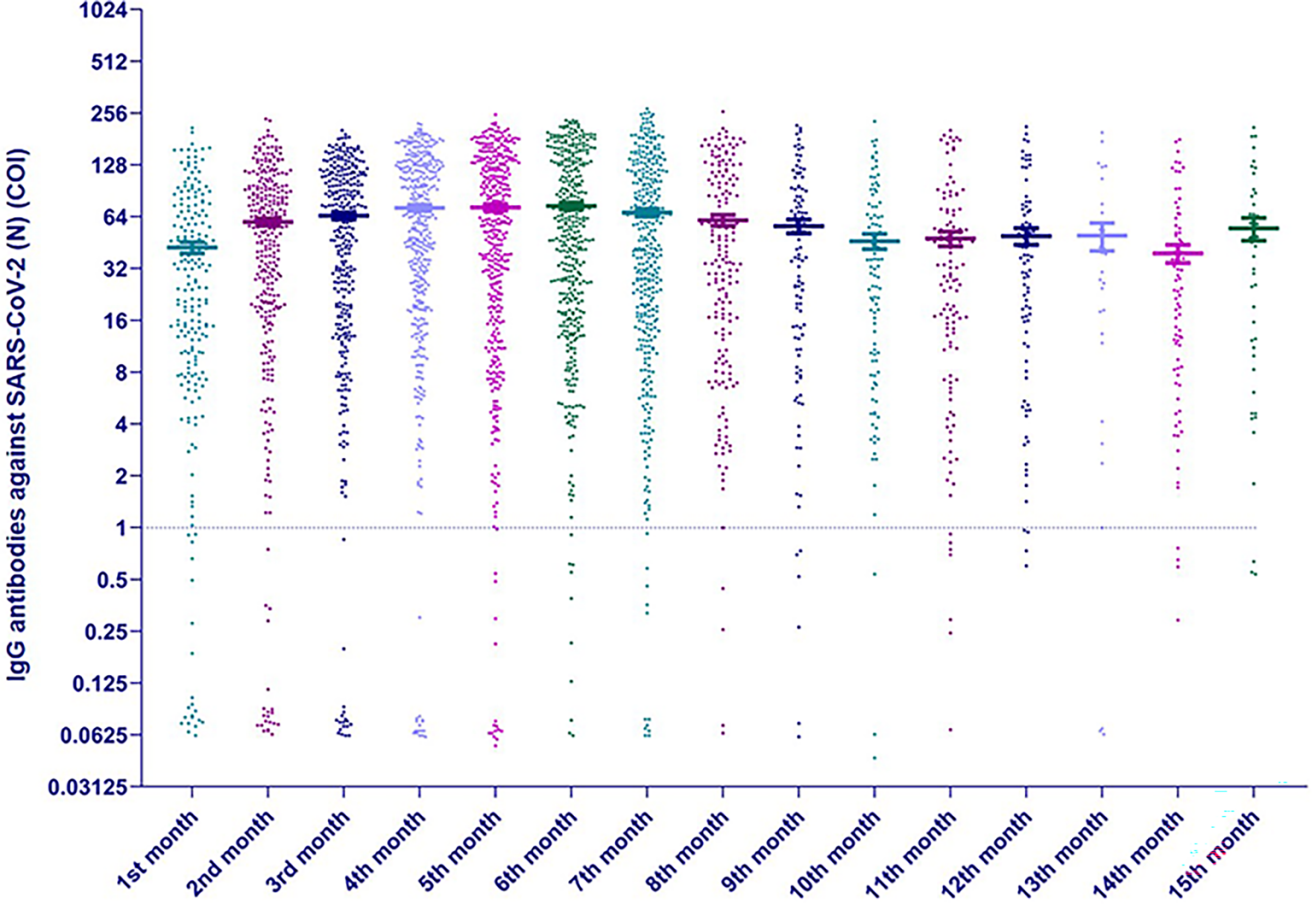
IgG antibodies titers against SARS-CoV-2 nucleocapsid protein (N) after the positive PCR testing every month (medians with ranges in logarithmic expression).

The level of anti-SARS-CoV-2 (N) IgG gradually increased up to 5–6th months and the decline of the antibody level starts from 7th month, nevertheless, the mean level remains rather high up to 15th month (Fig. 3). It is significant to mention that even in the group of convalescent patients who were tested after 52 weeks (up to 77 weeks), 95.3% of 211 samplings were still positive. Over the entire 18-week period of follow-up (5–54 weeks), only in 12 patients (1.7%) from 694 convalescent patients who were sampled at least 3 times, initially existing antibodies became undetectable (Supplementary Fig. 4).

89 convalescent patients (6.2%), did not produce detectable levels of anti-SARS-CoV-2 (N) IgG during the consecutive measurements.

Analyzing the association of age, sex, disease severity and BMI with the presence of anti-SARS-CoV-2 (N) IgG among COVID-19 convalescent patients, the younger age (43.4 ± 15 years in seronegative vs. 48.1 ± 15 in seropositive; *p* = 0.0028), the male sex (34% in seronegative vs. 25% in seropositive; *p* = 0.0381), less severe disease (moderate and severe disease in 13% in seronegative vs. 65% in seropositive; *p* < 0.0001) and the less BMI (25.5 ± 4.5 vs. 27.2 ± 5.2 in seropositive; *p* = 0.008) were significantly associated with the probability to be seronegative for anti-SARS-CoV-2 (N) IgG. In multivariate analysis only, disease severity was significantly associated with the probability to develop anti-SARS-CoV-2 (N) IgG with odds ratio 0.31 (0.17; 0.59) (*p* = 0.004) (Table 2).

**Table 2:** Association of age, sex, disease severity and BMI with the presence of anti-SARS-CoV-2 (N) antibodies among COVID-19 convalescent patients.

	IgG antibodies against SARS-CoV-2 (N) protein titer < 1	IgG antibodies against SARS-CoV-2 (N) protein titer > 1	*P* value
	n = 105	n = 1336	
Age (mean ± SD)	43.4 ± 15.1	48.1 ± 15.1	0.0028
**Sex (patients number and prevalence)**			
Male	36 (34%)	335 (25%)	0.0381
Female	69 (66%)	1000 (75%)	
**Severity**			
Asymptomatic	13 (12%)	91 (7%)	< 0.0001
Mild	78 (74%)	768 (57%)	
Moderate	12 (11%)	417 (31%)	
Severe	2 (2%)	60 (4%)	
BMI (mean ± SD)	25.5 ± 4.5	27.2 ± 5.2	0.0076

**Figure 4:**
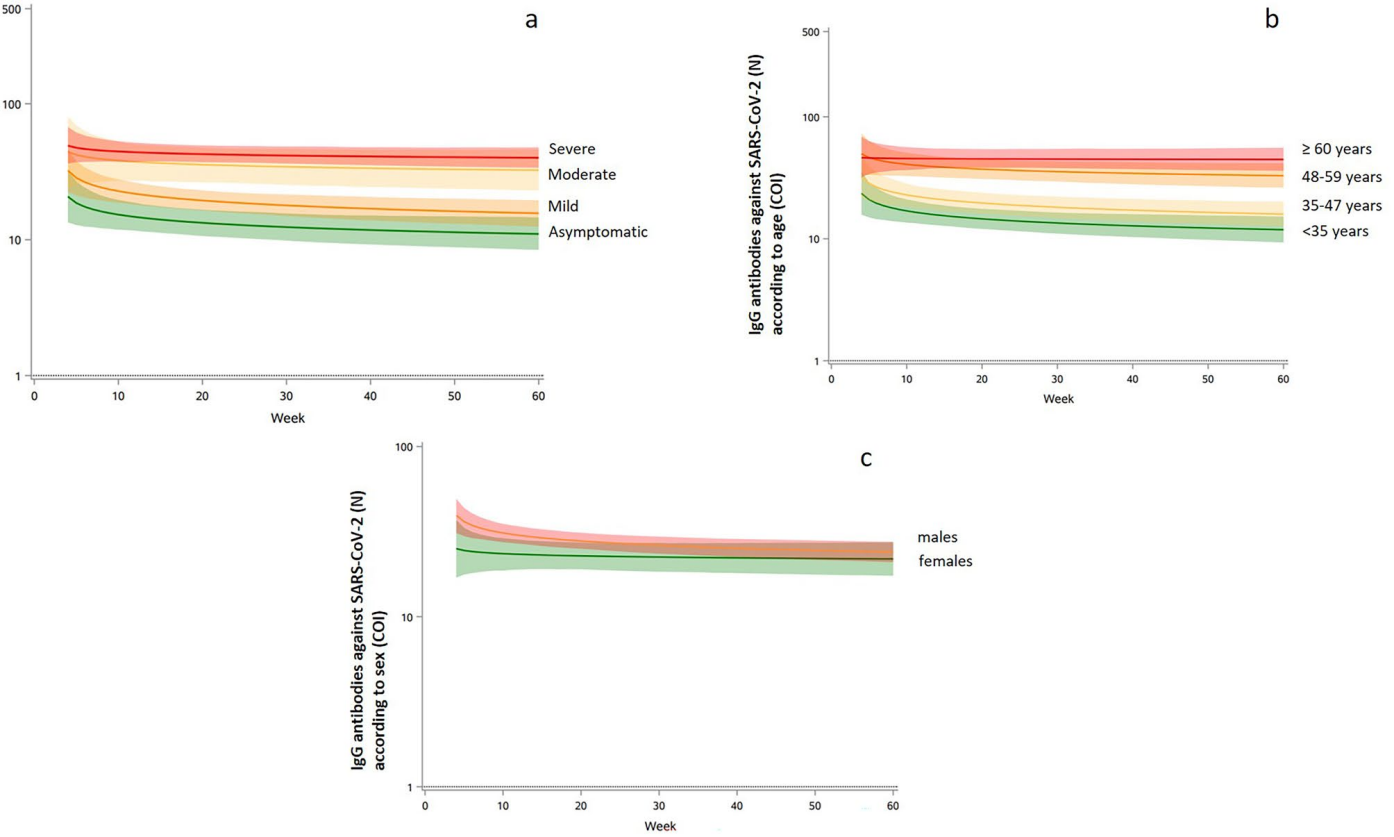
IgG antibodies kinetics against SARS-CoV-2 nucleocapsid protein (N) according to the disease severity, age and sex. (**a**) The kinetics of IgG antibodies against SARS-CoV-2 nucleocapsid protein (N) in asymptomatic, mild, moderate, and severe patients' groups. (**b**) The kinetics of IgG antibodies against SARS-CoV-2 nucleocapsid protein (N) according to age within < 35; 35–47, 48–59, ≥ 60 groups. (**c**) The kinetics of IgG antibodies against SARS-CoV-2 nucleocapsid protein (N) antibodies in males (green) and females (pink).

**Figure 5:**
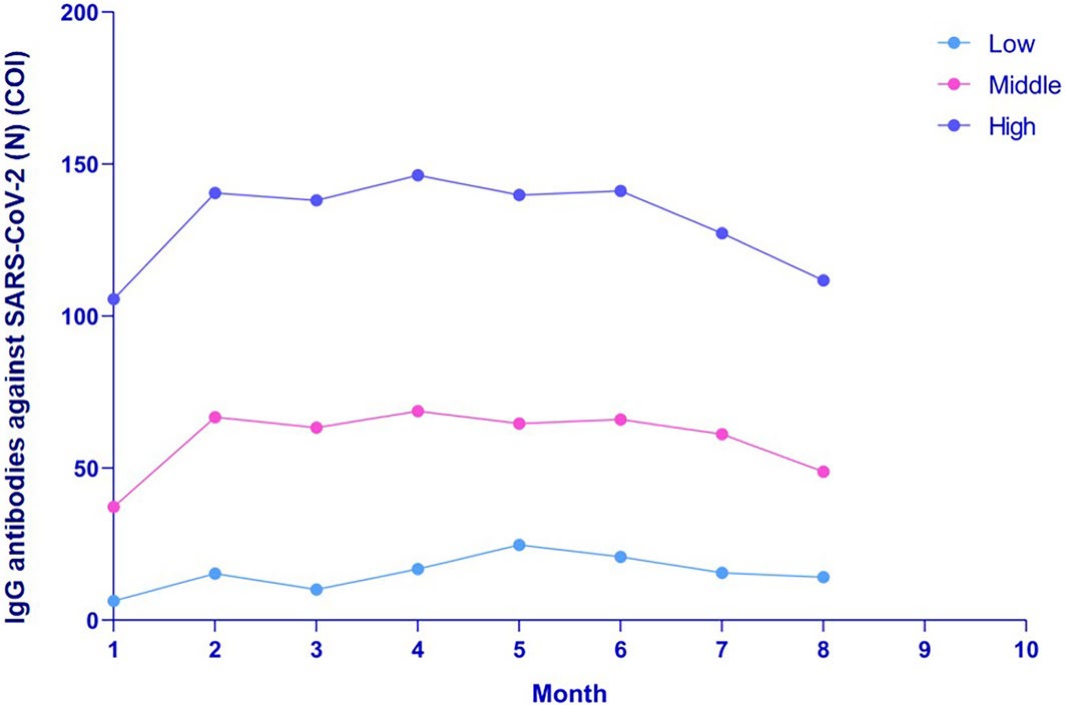
IgG antibodies against SARS-CoV-2 nucleocapsid protein (N) in low, middle and high responders.

To describe the anti-SARS-CoV-2 (N) IgG kinetics and correlate to disease severity, age, sex and BMI, we used a mixed statistical model which calculates the peak of the antibodies response and then a decay rate during the weeks after the peak^20^. The peak of antibodies response was estimated to 35COI (95% CI 29; 42), with decay rate at 1.11COI by week (95% CI 1.04; 1.17). Regarding disease severity and age, moderate and severe disease and more than 48 years old patients have higher levels of antibodies decay, whereas sex did not affect differently (Fig. 4a–c). Among factors associated with anti-SARS-CoV-2 (N) IgG kinetics, peak of antibodies were significantly higher in females, moderate and severe SARS-CoV-2 disease and aged more than 60 years (Table 3), whereas the decay rates were not significantly different (Figs. 4a–c). As the disease severity was significantly correlated with age (*p* < 0.0001), we analyzed anti-SARS-CoV-2 (N) IgG kinetics in patients groups considering the median age and the disease severity (age < 48 years with asymptomatic and mild COVID-19 infection and those ≥ 48 years and moderate to severe infection), demonstrating a significant correlation with only age (*p* = 0.0089) and not disease severity when adjusted to age (*p* = 0.22). Anti-SARS-CoV-2 (N) IgG titers of samples during the first month after a positive PCR were considered according to 25th and 75th percentiles and thus classified in low responders (titers of IgG up to 25th percentiles; n = 24), middle responders (titers of IgG 25–75 percentiles; n = 53) (COI value 10–75) and high responders (n = 24) (titer above 75th percentile; COI value above 76) (Fig. 5). Interestingly, anti-SARS-CoV-2 (N) IgG titers remained in the same levels in all these 3 groups during the subsequent monthly testings.

**Table 3:** Factors associated with the kinetics of anti-SARS-CoV-2 nucleocapsid protein (N) IgG antibodies.

	Anti-SARS-CoV-2 (N) antibodies peak (k, log10 COI)) (95%Q)	*P* value	Anti-SARS-CoV-2 (N) antibodies decay rate (a, (log10 COII)) (95%Q)	*P* value
Sex (male)	1.40 (1.24; 1.56)	ref		ref
Sex (female)	1.60 (1.50;1.69)	0.042	0.051 (0.024;0.082)	0.21
**Severity**				
Asymptomatic	1.45 (1.16;1.74)	ref	0.063 (− 0.026;0.152)	ref
Mild	1.42 (1.32;1.53)	0.86	0.059 (− 0.027;0.91)	0.93
Moderate	1.78 (1.62;1.94)	0.053	0.015 (− 0.035;0.64)	0.35
Severe	1.81 (1.41;2.21)	0.16	0.035 (− 0.086;0.156)	0.71
**Age**				
< 35 years	1.37 (1.20;1.55)	ref	0.074 (0.019;0.129)	ref
35–47 years	1.50 (1.33;1.67)	0.30	0.074 (0.021;0.126)	0.99
48–59 years	1.70 (1.52;1.87)	0.0097	0.044 (0.008;0.0.96)	0.45
≥ 60 years	1.67 (1.50;1.84)	0.018	0.004 (0.048;0.055)	0.068
Age < 48 years and asymptomatic -mild disease	1.31 (1.12;1.50)	ref	0.066 (0.008;0.125)	ref
Age ≥ 48 years and asymptomatic -mild disease	1.65 (1.39;1.91)	0.042	0.034 (− 0.046;0.114)	0.52
Age ≥ 48 years and moderate—severe disease	1.69 (1.56;1.83)	0.0015	0.022 (− 0.019;0.0.63)	0.23

Individual antibody data measured during the 15-month study period were modeled using a power law model, given by: f (t) = k − a log (c + t) (1), where f (t) is the log antibody titer at time of post infection (starting from t0 = 4 weeks), k is the peak log level, a is the decay rate, and c is an arbitrary small constant (set to 1).

## Discussion

In this large study from a middle-developed European country, have been analyzed one branch of the long-term humoral response to SARS-CoV-2 infection, in particular, anti-SARS-CoV-2 (N) IgG and the factors associated with a durable response. Based on an extensive literature review, it is noticeable that this study is one of the most long-drawn and broad studies of the dynamic changes in anti-SARS-CoV-2 (N) IgG in convalescent SARS-CoV-2 patients. There is demanding importance to explain the robustness, the survival, and the functionality of anti-SARS-CoV-2 (N) IgG response according to disease severity to discover the durability and protective features of antibodies in case of reinfection.

The condition that logical 15-month cohort study prompts us to make several important deductions about the anti-SARS-CoV-2 (N) IgG mediated humoral response after SARS-CoV-2 infection. Data obtained from our study clearly shows that SARS-CoV-2 infection induces a rapid humoral response mediated via anti-SARS-CoV-2 (N) IgG in almost all infected patients (92.7%), whereas only few patients developed a delayed humoral response (1.3%). Another study analyzing a shorter 180-day serological response to SARS-CoV-2 infection showed a relatively good humoral response, with much less seronegative patients and more proportion of delayed responses^21^. In a small percentage of convalescent patients (1.7%) the antibodies were not detectable during the 15 month follow-up, relatively similar to a previous study of 123 infected patients^19^, however, followed-up only for 30 weeks.

Our findings postulated that the level of anti-SARS-CoV-2 (N) IgG staked bell-shaped character, continuously growing up to 5–6 months, remaining stable for a few months and slowly decreasing, but remaining to be detectable up to 60 weeks. It is interesting, that initially different levels of induced SARS-CoV-2 antibodies remain stable at their levels during the 15-month follow-up. Based on the report of Nag et al. (2020) and Long et al. (2020) anti-SARS-CoV-2 (N) IgG degrade quickly over one to 3 months, possibly resulting in reinfections^10,11^. According to Dispinseri et al. (2021), the titer of neutralizing antibodies dwindled rapidly after 5–8 weeks^22^. In contrast, our investigation showed the stability of anti-SARS-CoV-2 (N) IgG up to 25–30 weeks. Data similar to our results showing the increase of antibody levels were in another study analyzing 5 month humoral response Fotouhi et al. (2021)^23^.

Regardless of the initial level of IgG, antibody production increases in the first stages. Depending on the point of departure in terms of anti-SARS-CoV-2 (N) IgG, the "future journey" of antibodies differs. Based on the anti-SARS-CoV-2 (N) IgG titer, we divided convalescent patients into 3 groups: low, middle and high responders. The findings affirm the hypothesis that the magnitude of antibody response in high responders remains higher throughout the whole period of antibody generation compared to low and middle responders; even we did not assess in this study their correlation with their functionality or avidity.

According to various authors, the persistence of antibodies depends on many factors, including the viral type^24^, individual features, and environmental factors. This explains the many varying courses of the disease and the corresponding immune responses in individuals.

Analyzing the seropositive and seronegative SARS-CoV-2 convalescent patients, the severe form of the disease appeared as an independent factor to develop anti-SARS-CoV-2 humoral durable response. Several studies in other viral diseases, and also those including severe SARS-CoV-2 infected cases showed higher levels of anti-SARS-CoV-2 humoral response in more severe disease^1,12,14,24,25^.

Another meaningful quest in the SARS-CoV-2 research is to discover correlations of the immune response with various individual factors such as age, sex or disease severity. In an Italian study, of healthcare professionals, a higher prevalence of positive IgG was found in females^26^. BMI did not influence the frequency of IgG-positivity in individuals, but it was directly proportional with the plasma concentration. In older patients (> 60 year), the frequency of IgG positivity drops, but when assessing the difference of IgG plasma levels across age ranges, an increased level of plasma IgG with older age is found^26^. Few studies found more important early increase of anti-SARS-CoV-2 (N) IgG in men, but later in the disease the antibody levels were equal between sexes. In some studies, no association or even negative association between BMI and anti-SARS-CoV-2 (N) IgG was observed^16,27^. In contrary, we can affirm that the levels of SARS-CoV-2 humoral response, in particular the anti-SARS-CoV-2 (N) IgG peak level, were significantly more important in older patients, regardless of their sex, disease severity and BMI.

The capability of reacting to infections decaying with age is a well-known fact. Besides the change of the functional types of T and B cells and the immune balance in the aging population, fewer cells able to identify and fight against new infections are produced with the age^28,29^.To form a completely new immune response to a novel infection is one of the weakened capacities of the elderly. The basis for this is the decline of naïve T cells which are required to start an entirely new immune response due to the shrinking of the thymus with age^30^. We hypothesized that the age-related changes to T cell immunosenescence can be the reason for the compensator increase of humoral immune response and antibody production in older individuals during the COVID-19. On the other hand, there are studies that shown the slower generation and lower virus neutralizing capacity of antibodies against attenuated yellow fever virus vaccine compared with the young population^29^. Therefore, we suggest that the high titer of antibodies in the elderly is compensation of the lower virus neutralizing capacity and low affinity of antibodies.

Several limitations of the current study should be noted. Our study was focused on humoral axis of immune response, wherein the results were limited to detecting only anti-SARS-CoV-2 (N) IgG using a semi-qualitative test. Other parts of the humoral response as SARS-CoV-2 spike protein, neutralizing antibodies, anti-RBD/S antibodies, as well as nuances like individual avidity and affinity were not evaluated in this study and needed further investigations. It is also important to thoroughly analyze and compare antibody response profile in both asymptomatic and symptomatic cases of COVID-19.

## Conclusion

Summing up, we found that the kinetics of anti-SARS-CoV-2 (N) IgG took a bell-shaped character and has been still detectable for more than 15 months. Collectively, our data demonstrates that on the one hand anti-SARS-CoV-2 humoral response level correlates with disease severity, on the other, in particular, the level of peak antibodies correlates with age – older patients develop more robust humoral response regardless of sex, disease severity and BMI.

## Methods

### Samples

In this prospective nationwide study, 1441 consecutive SARS-CoV-2 convalescent patients were recruited from all 10 regions of Armenia, and capital Yerevan city from August 2020 to June 2021. The inclusion criteria were (i) recent SARS-CoV-2 infection (compatible clinical features with positive RT-PCR result on nasopharyngeal swab samples), (ii) convalescent patients without any clinical symptoms equivocal of SARS-CoV-2 infection at the time of samples collection and (iii) absence of any induced or inherited immunodeficiency (HIV infection, neoplasia, hematological diseases, or immunosuppressive therapies). The exclusion criteria were pregnancy, age < 18 years, clinical sign of COVID-19 in anamnesis with negative PCR or without PCR testing, presence of any primary or secondary immunodeficiency.

The appropriate confirmation of PCR testing was presented by patients in form of official document from laboratory where the testing was done.

Two groups of healthy donors were included in the study. The first, “pre-pandemic control group” consists of serum samples of 71 healthy donors before SARS-CoV-2 pandemic period (from 2017 up to February 2020), the second “SARS-CoV-2 pandemic group” consist of serum samples of 150 healthy donors throughout the SARS-CoV-2 pandemic period (started from March 2 of 2020).

SARS-CoV-2 convalescent patients were examined for anti-SARS-CoV-2 (N) IgG after 3–4 weeks of positive PCR testing, and then monthly up to 19 months (Fig. 1a–c). During the study inclusion period, 4266 serum samples of 1441 convalescent patients were collected at different times after SARS-CoV-2 infection (Fig. 1b,c).

The appropriate sample size for a population-based survey in Armenia is 384. In our study the number of participants during the 4th, 5th, 6th and 7th months overpassed this threshold. The donors of the “pre-pandemic control” and “SARS-CoV-2 pandemic” groups were sampled only once.

Patient’s general characteristics, SARS-CoV-2 infection features, comorbidities and were recorded at the time of first sample testing. After the analysis of clinical symptoms, patients were divided into 4 groups according to disease severity.

The disease severity was classified according to modified definitions of WHO^31^:

- Patients without any symptoms of COVID-19 with positive PCR testing were defined as asymptomatic.
- Patients meeting the case definition for COVID-19 without evidence of viral pneumonia or hypoxia were classified as mild cases.
- Clinical signs of pneumonia (confirmed via X-ray or CT-scan) with SpO2 ≥ 90% on room air was classified as moderate case, with SpO2 < 90%—severe case.

### Serological assay

The patients were sampled in Heratsi University Hospital Laboratory. Blood samples were taken from individual patient in laboratory by traditional venipuncture method in vacuum tubes with gel barrier. Afterwards, the collected blood samples were centrifuged for separating the serum from blood elements by standard protocol. Serum samples of each patient were analyzed for anti-SARS-CoV-2 (N) IgG immediately or undergo cold storage at 2–4 °C for 1–7 days. Sampling, transportation and destruction of the samples was done according to SOPs.

Serum samples of each patient were analyzed for in vitro detection of high-affinity IgG antibodies against full-length nucleocapsid (N) antigen of SARS-CoV-2with commercially available “**Elecsys**” assay from *Roche Diagnostics*. Based on producer instructions, results were automatically determined in the form of a cutoff index (COI), with COI quantitative values and COI < 1 were considered as negative, and ≥ 1 as positive result.

### Ethics

This study was conducted according to the principles of the Declaration of Helsinki and approved by the Ethics Committee of Yerevan State Medical University (N 8–2/20; 02.07.2020). Informed written consent was obtained from all participants.

### Statistical analysis

Statistical analysis was performed using the IBM_22.0.0 SPSS statistical package (IBM, Armonk, NY) and SAS v9.4 (SAS Institute, Cary, NC). Continuous variables with normal distribution were presented as means, standard deviation (SD), whereas categorical variables as numbers and percentages. The persistence of anti-SARS-Cov-2 levels over time was estimated using non-linear mixed effects models that estimate the peak and the rate of antibodies decay^20^. Timing of antibodies was determined from positive PCR date. Individual antibody data measured during the 15-month study period were modeled using a power law model, given by: f (t) = k − a log (c + t) (1) where *f *(*t*) is the log antibody titer at time of post infection (starting from t0 = 4 weeks), *k* is the peak log level, *a* is the decay rate, and *c* is an arbitrary small constant (set to 1). The models were fitted by a mixed effects method, where *k* and *a* are random effects, allowed to be patient specific and are assumed to be drawn from *a* bivariate normal distribution. This allowed a prediction of the antibody dynamics to be made for each person. Predicted results over time are reported as GMTs. Parametric bootstrap was used to estimate confidence intervals. A logistic regression model was used to derive multivariable-adjusted odds-ratio estimates of factors associated with a negative serology. All p values were from 2-tailed tests, and results were deemed statistically significant with *P* < 0.05.

## Supplementary Information

Supplementary Information 1.

Supplementary Information 2.

Supplementary Information 3.

Supplementary Information 4.

Supplementary Information 5.

## Data Availability

Data can be made available by the corresponding author upon reasonable request.
