# Supplementary figures and images for: Kinetics of anti-nucleocapsid IgG response in COVID-19 immunocompetent convalescent patients
Source: Sci Rep. 2022 Jul 20;12:12403. doi: 10.1038/s41598-022-16402-0 (PMC9297274; doi:10.1038/s41598-022-16402-0)

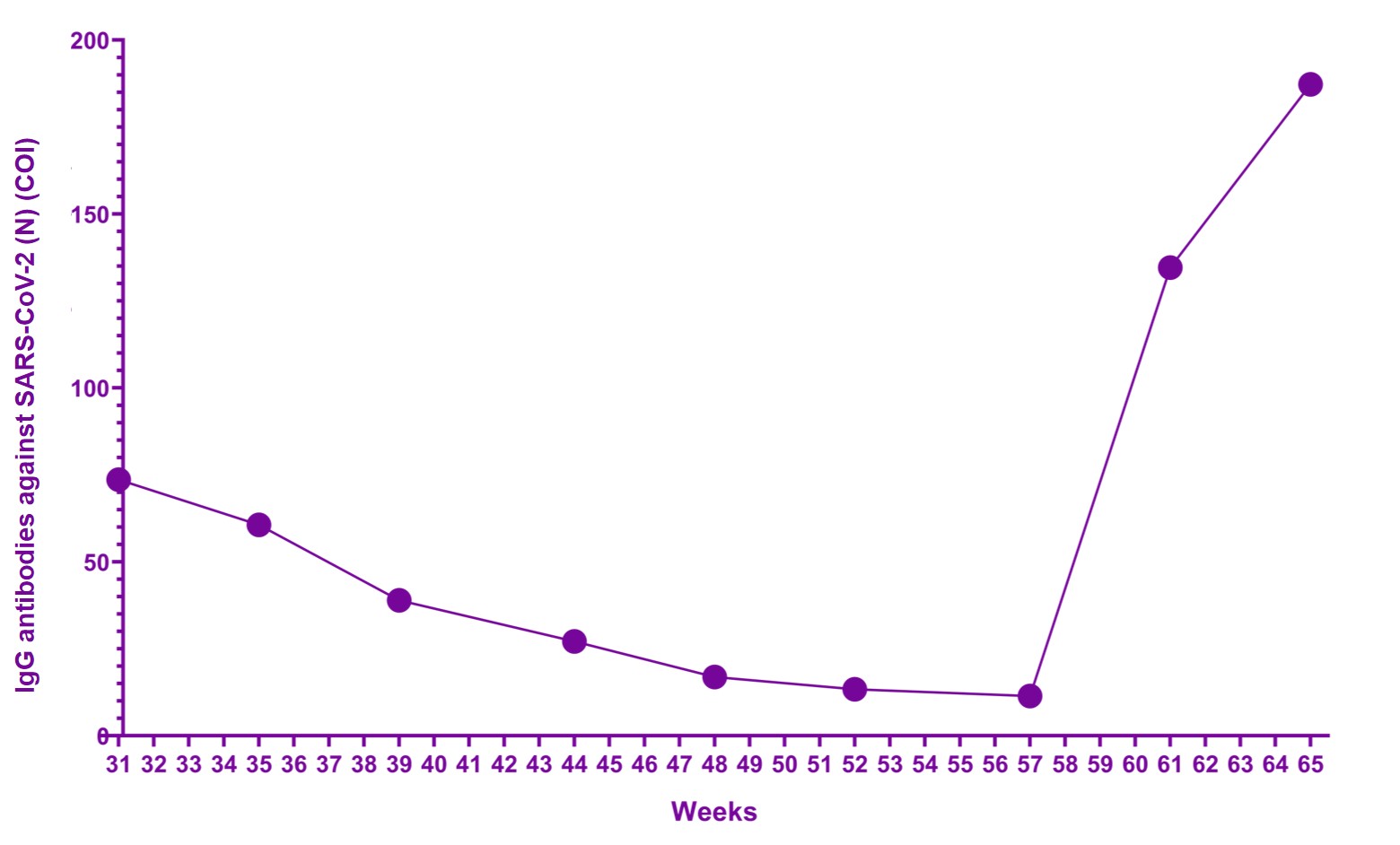

Supplement: Supplementary file 1 — Supplementary Information 1. [file 41598_2022_16402_MOESM1_ESM.jpg]

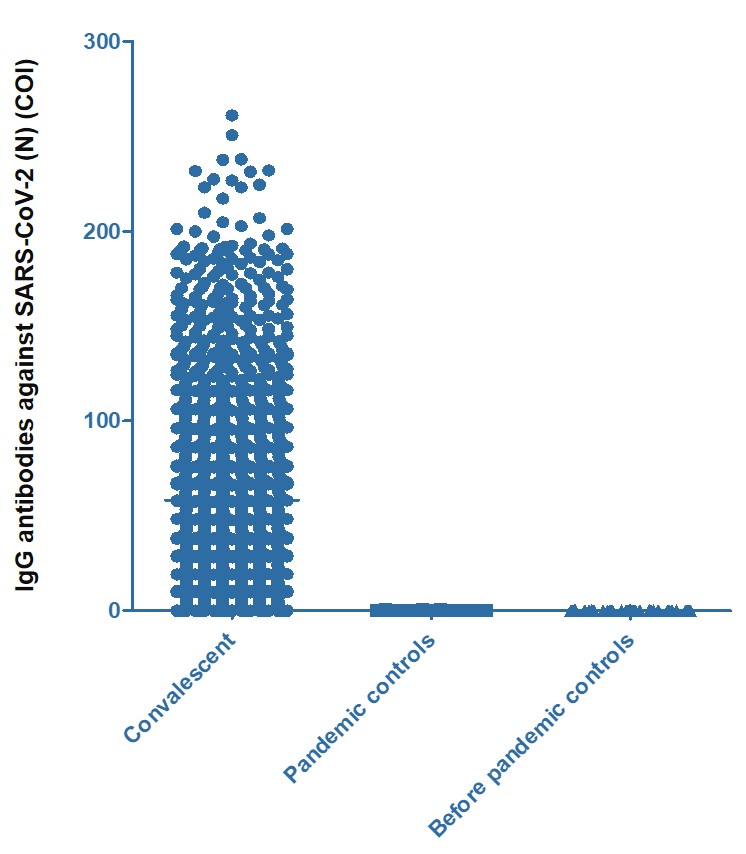

Supplement: Supplementary file 2 — Supplementary Information 2. [file 41598_2022_16402_MOESM2_ESM.jpg]

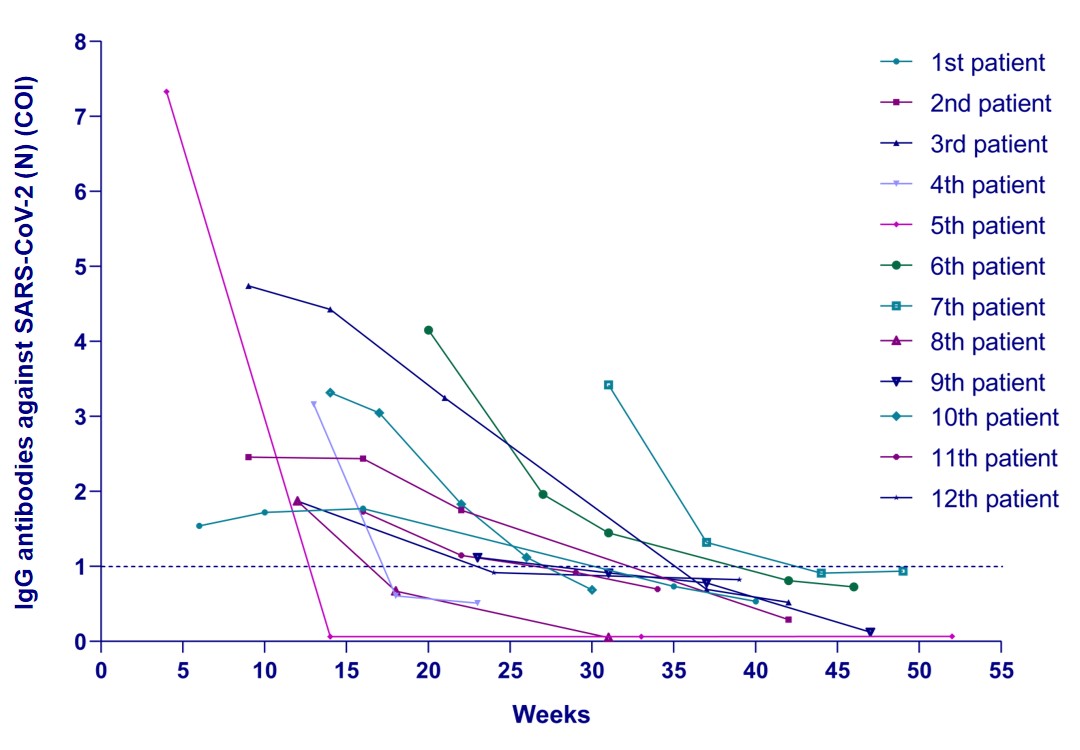

Supplement: Supplementary file 3 — Supplementary Information 3. [file 41598_2022_16402_MOESM3_ESM.jpg]

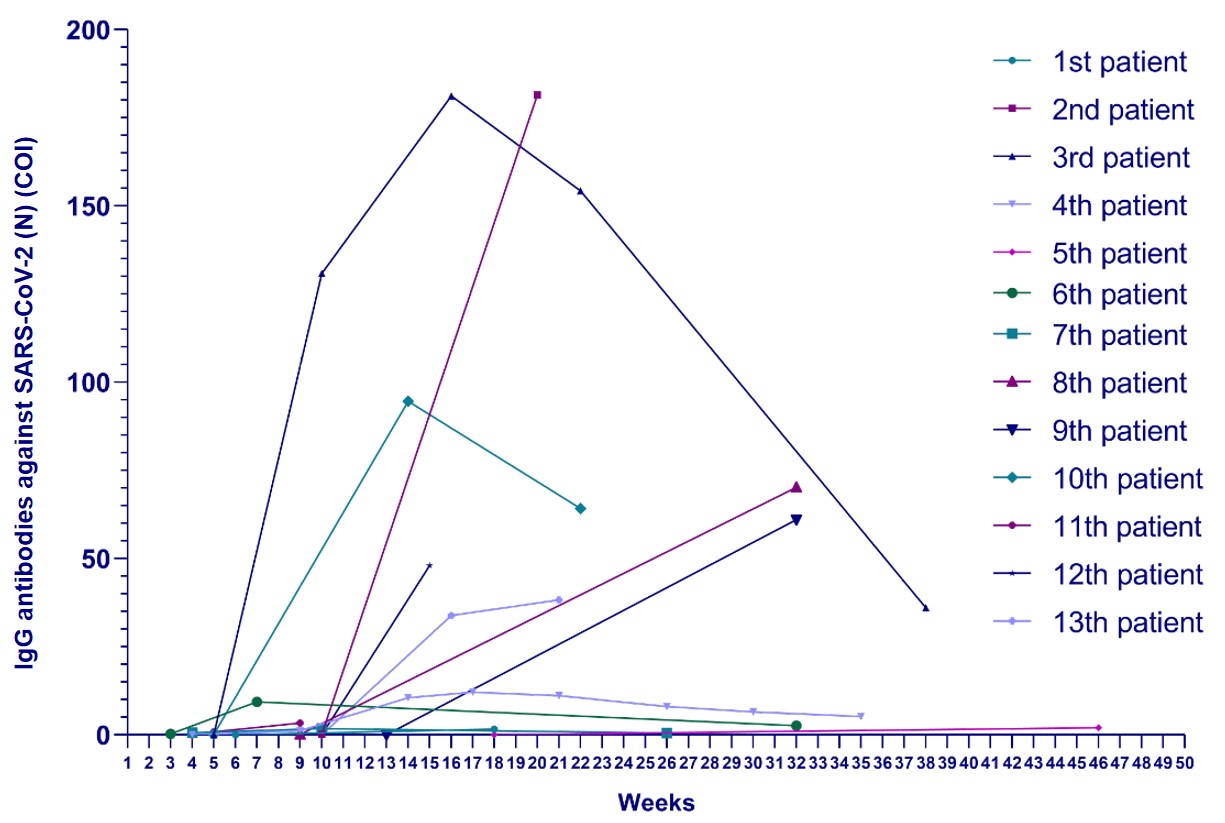

Supplement: Supplementary file 4 — Supplementary Information 4. [file 41598_2022_16402_MOESM4_ESM.jpg]
