# Supplementary material for: Kinetics of anti-nucleocapsid IgG response in COVID-19 immunocompetent convalescent patients
Source: Sci Rep. 2022 Jul 20;12:12403. doi: 10.1038/s41598-022-16402-0 (PMC9297274; doi:10.1038/s41598-022-16402-0)
Supplement: Supplementary file 5 — Supplementary Information 5. [file 41598_2022_16402_MOESM5_ESM.docx]

**Supplementary Figure 1. Reinfection case 12 months after the first positive PCR testing from the convalescent cohort.**

**Supplementary Figure 2. The levels of IgG antibodies against SARS-CoV-2 nucleocapsid protein (N) in convalescent patients in comparison to pre-pandemic and pandemic healthy controls.**

**Supplementary Figure 3.** Individual lines of anti-SARS-CoV-2 (N) IgG changes in 13 patients with delayed response.

**Supplementary Figure 4.** **Individual survival of anti-SARS-CoV-2 (N) IgG antibodies in patients whose initially existing antibodies disappeared.**

The anti-SARS-CoV-2 (N) IgG levels disappeared in 12 patients (1.7%) from 694 convalescent patients with initially existing antibodies.
